# Comparison of the Retention of Aliphatic Hydrocarbons with Polar Groups in RP-HPLC Systems with Different Modifiers of the Binary Eluent

**DOI:** 10.1007/s10337-013-2489-4

**Published:** 2013-06-09

**Authors:** Anna Klimek-Turek, Beata Misiołek, Tadeusz H. Dzido

**Affiliations:** Department of Physical Chemistry, Medical University of Lublin, Chodźki 4a, 20-093 Lublin, Poland

**Keywords:** Reversed phase liquid chromatography, Relative retention change, Modifier selectivity, Aliphatic hydrocarbons with polar groups

## Abstract

The retention of aliphatic hydrocarbons with polar groups has been compared in respect to the separation selectivity changes in reversed-phase high-performance liquid chromatography with C18 stationary phase type and binary water eluent composed of methanol, acetonitrile, or tetrahydrofuran as modifiers. The changes in separation selectivity when one modifier is replaced by another in the eluent is explained, taking into consideration molecular interactions of the solutes with components of the stationary phase region, i.e., extracted modifier, and ordering of the stationary phase by the modifier.

## Introduction

Wide range of stationary phases can be used in order to improve separation selectivity in contemporary high-performance liquid chromatography (HPLC). However, replacement of the HPLC column leads to discontinuous changes of retention and separation selectivity. This obstacle can be easily circumvented by changing the mobile phase composition. Tuning the retention and separation selectivity of analytes by matching the qualitative and quantitative composition of a mobile phase is the important advantage of HPLC. In spite of over 40 years' development of HPLC [1, 2] and progress in computer simulation of its process [3, 4], also based on molecular interactions [5], some aspects of the chromatographic process raise questions, particularly those concerning prediction of the retention and improvement of separation selectivity of solutes in reversed-phase systems. Therefore, a simple approach that enables interpretation of retention changes and putting forward clear conclusions is much desired. Such an approach was proposed earlier to explain the changes in separation selectivity of aromatic hydrocarbons with polar groups [6] and phenolic acids [7, 8]. It assumes that selectivity changes generated by the change of the modifier in the eluent can be explained taking into consideration only molecular interactions between the solute and modifier in the stationary phase (interactions in the mobile phase are neglected) and also taking into account ordering of the stationary phase by the modifier [6, 9]. In this article, we discuss relative retention changes of aliphatic hydrocarbons with different polar groups that confirm the postulated approach based on the previously published data and recent experiments [6–11]. The following discussion of the relative retention changes of the solutes is based on their abilities in molecular interactions with modifier molecules extracted into the stationary phase of reversed phase high-performance liquid chromatography (RP-HPLC) systems. The abilities of the modifier molecules in these interactions are expressed in a solvent selectivity triangle, proposed by Snyder and modified using normalized solvatochromic Kamlet-Taft parameters [12]. The substances of interest, aliphatic hydrocarbons with polar groups, have the smallest molecular volume among the compounds tested so far, so even subtle differences in the construction of their molecules can affect their retention and selectivity. Investigations of liquid chromatography of aliphatic compounds have been documented in a few papers. Wang et al. [13] studied their retention in reversed-phase liquid chromatography using linear solvation energy relationships (LSER). The LSER coefficients were then examined in terms of corresponding properties of the mobile phase (cohesive energy density, surface tension, the Abraham solvophobic parameter, polarity/polarizability, hydrogen bond basicity, and hydrogen bond acidity), and from these, the influence of the mobile phase and stationary phase on retention behavior was explored [13, 14, 16].

Our study focuses on the interpretation of the selectivity changes of aliphatic compounds with polar groups when an organic modifier is replaced by another one in the RP-HPLC system. The solvation parameter model proposed by Abraham was used as a comparative tool to confirm and explain the selectivity changes between systems with various modifiers.

## Experimental

Solutes (Table 1) were obtained from different sources. All solvents and chemicals were of analytical grade. Water was bidistilled. Measurements of retention were performed with an HP 1050 liquid chromatograph (Hewlett-Packard, Wilmington, DE, USA) equipped with a 20-μL sample injector (Rheodyne, Cotati, CA, USA) and a refractive index detector type RIDK-102 (Laboratorni Pristoje Praha).

**Table 1:** List of solutes investigated and their descriptors [22]

No.	Name	*A*_H_ hydrogen bond acidity	*B*_H_ hydrogen bond basicity	*S* dipolarity	*V* molecule volume
1	Butan-1-ol	0.37	0.48	0.42	0.73
2	2-Methylpropan-1-ol	0.37	0.48	0.39	0.73
3	Pentan-1-ol	0.37	0.48	0.42	0.87
4	3-Methylbutan-1-ol	0.37	0.48	0.39	0.87
5	2-Methylbutan-2-ol	0.31	0.53	0.36	0.87
6	Hexan-1-ol	0.37	0.48	0.42	1.01
7	Cyclohexanol	0.32	0.57	0.54	0.90
8	Pentan-2-one	0	0.51	0.68	0.83
9	3-Methylbutan-2-one	0	0.51	0.65	0.83
10	Hexan-2-one	0	0.51	0.68	0.97
11	4-Methylpentan-2-one	0	0.51	0.65	0.97
12	Propyl acetate	0	0.45	0.60	0.89
13	1-Methylethyl acetate	0	0.47	0.57	0.89
14	Butyl acetate	0	0.45	0.60	1.03
15	2-Methylpropyl acetate	0	0.47	0.57	1.03
16	Methyl butanoate	0	0.45	0.60	0.89
17	Methyl-2-methylpropanoate	0	0.47	0.57	0.89
18	1-Propoxypropane	0	0.45	0.25	1.01
19	2-Propan-2-yloxypropane	0	0.41	0.19	1.01
20	1-Nitropropane	0	0.31	0.95	0.71
21	2-Nitropropane	0	0.33	0.92	0.71

Chromatography was performed using a stainless-steel column (4.6 × 100 mm), which was packed with Lichrosorb RP-18 (C18 type), Si 100 (Merck KGaA, Darmstadt, Germany), after silanization with bis(trimethylsilyl)amine. Specification of stationary phase: coverage density 3 μmol m^−2^, surface area 300 m^2^ g^−1^, and carbon load 16.2 %. The hold-up volume was determined for each mobile phase used by the injection of a sample of pure water. The mobile phase contained 0.1 % acetic acid for suppressing dissociation of the residual silanol groups. The chromatographic experiments were carried out at 22 ± 0.1 °C.

## Result and Discussion

### Acetonitrile (ACN) versus Tetrahydrofurane (THF)

**Fig. 1:**
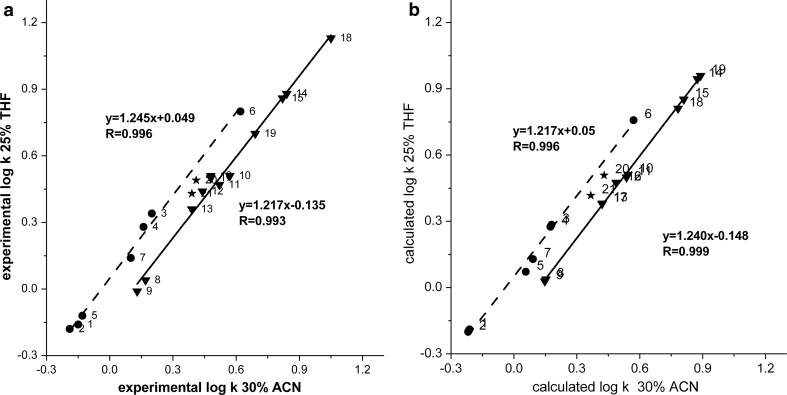
**a** Correlation of experimental log*k* values of aliphatic hydrocarbons with polar functional groups for the systems with 30 % ACN and 25 % THF; *triangles* compounds with electron-donor groups, *circles* aliphatic alcohols; solute numbers in Table 1. **b** Correlation of calculated log*k* values of aliphatic hydrocarbons with polar functional groups for the systems with 30 % ACN and 25 % THF; *triangles* compounds with electron-donor groups, *circles* alcohols; solute numbers as in Table 1

The results of the investigations are demonstrated in the figures as correlations of log*k*_1_ vs. log*k*_2_ where *k*_1_ and *k*_2_ are retention factors of the solutes in systems 1 and 2, respectively. In Fig. 1a, retention of the solutes in 25 % THF against 30 % ACN with the C18 stationary phase is correlated. The solutes form two separate lines—one for substances with proton donor groups and the other for substances with electron donor groups only. The correlation line for aliphatic alcohols (dashed line), solutes with the proton-donor and proton-acceptor interaction ability, is above the line for solutes with electron-donor (proton acceptor) groups (solid line). This indicates relatively higher retention of aliphatic alcohols relative to aliphatic derivatives with electron-donor groups in the system with THF in comparison to that with ACN. This effect is explained, according to our approach [6], by stronger hydrogen bond interactions of the aliphatic alcohol molecules with THF in the stationary phase region. Additionally, this effect is enhanced by greater sorption of THF than ACN in the stationary phase, especially when the mobile phase is water-rich [14, 15]. Furthermore, investigated solutes with proton acceptor groups (except for 1-propoxypropane and 2-propan-2-yloxypropane) show higher dipole moment values than aliphatic alcohols (Table 1), which also may enhance their molecular interactions with ACN relative to THF in the stationary phase. Although the dipolar interaction ability of ACN and THF is similar according to the values of solvatochromic parameters, the electric dipole moment of the former modifier is almost two times greater than that of the latter (3.45 and 1.75 D for ACN and THF, respectively) [18, 19].

Figure 1b demonstrates the correlation of the retention parameter, log*k*, calculated using multiple linear regression. The calculations are based on Abraham’s equation [20, 21]:

1 $$ \log k = a_{\text{H}} \sum A_{\text{H}} + b_{\text{H}} \sum B_{\text{H}} + sS + eE + vV + c $$

where *k* is the retention factor; *A*_H_ and *B*_H_ are the hydrogen bond acidity and basicity, respectively; *S* is the dipolarity/polarizability; *E* is the excess molar refraction; *V* is the McGovan volume; *c* is the intercept; the *a*_H_, *b*_H_, *s*, *e*, and *v* coefficients characterize the respective properties of the stationary phase/mobile phase system.

The coefficients of the systems with 40 % MeOH, 30 % ACN, and 25 % THF are presented in Table 2.

**Table 2:** The values of coefficients characterizing the stationary phase/mobile phase systems

	*a*	*b*	*s*	*v*	*e*	*c*
MeOH	0.3679	−2.0747	−0.0914	3.2480	0.1526	−1.4214
ACN	−0.4684	−2.9570	0.1083	2.7835	0.1907	−0.7401
THF	−0.0299	−4.1833	0.1787	3.3744	0.3315	−0.7819

In Fig. 1b, solute points forming two correlation lines, analogous to those shown in Fig. 1a, can be observed. This means that the calculated and experimental group selectivities are similar. The correlation lines have nearly the same slope. The difference between intercepts of the parallel lines is mainly determined by the different hydrogen-bonding energy values of each series. Based on the values of parameters *a* and *b* (relative basicity and acidity of the systems, respectively), one can notice that the *b* coefficient of the THF system is more negative than that of the ACN system, while the *a* coefficient demonstrates the reverse relation. This indicates that the THF system shows weaker hydrogen bond acidity than the ACN system. On the other hand, the THF system demonstrates a stronger basic property of the hydrogen bond than the ACN system. This confirms that solutes with a hydrogen donor ability show a relative retention increase compared to those with a hydrogen donor acceptor ability in THF system compared to the ACN system.

If solutes demonstrate similar values of proton-donor and proton-acceptor abilities, but differ in size and shape, then the ordering of the stationary phase region by organic modifier molecules will affect additional changes of their separation selectivity [23]. The values of the separation factor, *α*, for the structural isomer pairs are presented in Table 2. Differences in the values of the parameter *α* for solute pairs in the system with THF in comparison to the system with ACN, e.g., pentan-2-one and 3-methylbutan-2-one (*α*_THF_ = 1.12, *α*_ACN_ = 1.08), propyl acetate, and 1-methylpentan-2-one (*α*_THF_ = 1.20, *α*_ACN_ = 1.13), were evident. This means that retention of *n*-isomers is increased relative to that of iso-isomers in the THF system in comparison to the ACN one. This effect is presumably concerned with slightly lower entropic penetration into the more strongly ordered stationary phase region of the THF system relative to the less ordered stationary phase region of the ACN system by solute molecules of branched structure (iso-isomer) in comparison to those of less branched structure (*n*-isomers). Based on calculated retention data, the analogous values of the *α* parameter for pentan-2-one and 3-methylbutan-2-one, and propyl acetate and 1-methylpentan-2-one are: 1.02 and 1.03 for the ACN and the THF systems, respectively. These values are very close, which is contrary to the experimental data mentioned above. This relationship can be explained by the lack of a parameter representing the molecular shape in Eq. (1). In our opinion, such a parameter could better differentiate the calculated retention of branched molecules relative to less branched ones.

In the case of solute pairs such as hexan-2-one and 4-methylpentan-2-one, and butyl acetate and 2-methylpropyl acetate, the effect of the retention increase of *n*-isomers relative to their iso-isomers is considerably reduced in relation to analogous solutes with the propyl constituent discussed above. It means that the retention ratio of two isomers whose molecules are equipped with longer aliphatic constituents is decreased in comparison to that of two analogous isomers with a shorter non-polar constituent in the THF system relative to the ACN one. Such selectivity changes of two isomers with shorter *n*-aliphatic and iso-aliphatic constituents are larger in THF system than in the ACN system; however, this selectivity change is diminished if the aliphatic constituent is elongated. This means that branching of the short non-polar constituent of solute isomers more strongly impacts the separation selectivity in the THF system relative to the ACN system than the same branching of longer non-polar constituents of analogous isomers in the same modifier systems. The discussion above can be confirmed by the relative retention data obtained for pentan-1-ol isomers. The values of parameter *α* for pentan-1-ol and 3-methyl-1-butanol are 1.08 (30 % ACN) and 1.15 (25 % THF), respectively [one can notice a similar dependence for butan-1-ol and 2-methylopropan-1-ol: 1.04 (30 % ACN) and 1.11 (25 % THF), respectively]. The values of the separation factor for pentan-1-ol and 2-methylbutan-2-ol are 2.12 for the system with ACN and 2.88 for the system with THF. Based on these data, one can conclude that aliphatic alcohols of more branched molecular structures (e.g., *tert*- and *iso*-isomer) show decreased retention relative to *n*-alcohol in the THF system compared to the ACN system. It should be mentioned that this phenomenon could be additionally enhanced by decreasing the acidity of the hydrogen bond interaction of the *tert*-isomer because of its lower parameter *A*_H_ value. Generally speaking, the stationary phase in the THF system is more ordered and thus is less entropically accessible for branched molecules than that of the ACN system. A complementary effect, which supports the explanation of the discussed selectivity changes of aliphatic alcohols, may be concerned with different probabilities of hydrogen bond formation between various isomers of aliphatic alcohols and THF molecules in the ordered stationary phase region. It is reasonable that molecules with a more branched structure show a lower probability of H-bond interactions than those with *n*-alkyl chains, which are more flexible.

The next example of relative retention changes in the THF system in comparison to the ACN system is demonstrated for pairs of aliphatic esters and ketones. In Table 4, the values of the separation factor, *α*, for several pairs of aliphatic hydrocarbons with ester and carbonyl functional groups are presented. Substances combined in pairs have an equal number of carbon atoms, while one solute is an ester and the other is a ketone. The values of the separation factor of the solute pairs are greater in the THF system than in the ACN system.

Ketones have greater dipole moment values than esters (Table 1), which can lead to stronger dipolar interactions of the former solutes with the modifier in the stationary phase region of the ACN system than with the modifier in the stationary phase of the THF system. This can explain the discussed decrease of the relative retention of the solutes in the 30 % ACN system in comparison to the 25 % THF system. It should be mentioned that higher dipolar properties of the ketones relative to the esters coincide with their log*P* values, i.e., esters are less polar than ketones (log*P* is equal to 1.24 and 0.84 for propyl acetate and pentan-2-one, respectively; 1.78 and 1.38 for butyl acetate and hexan-2-one, respectively [27]). Hence, an increase of retention of the esters relative to the ketones in the THF system in comparison to the ACN system can be expected because the hydrophobic character of the stationary phase region with THF in the mobile phase is stronger than that with ACN. A similar effect can be observed for the following pairs of solutes: pentan-1-ol and butan-1-ol, 3-methylbutan-1-ol and 2-methylopropan-1-ol or hexan-1-ol andpentan-1-ol (Table 5). The separation factor values for these solute pairs are much higher for the THF system than for the ACN system. Relative retention changes of ethers are the next example of the discussed effect. The values of experimental parameter *α* for 1-propoxypropane and 2-propan-2-yloxypropane are 2.30 and 2.72 for the 30 % ACN and 25 % THF systems, respectively. Based on these values, one can estimate that an ether isomer of more hydrophobic molecules, e.g., 1-propoxypropane (log*P* = 2.03), shows a retention increase relative to the less hydrophobic one, e.g., 2-propan-2-yloxypropane (log*P* = 1.5), in the THF system in comparison to the ACN system. On the other hand, the calculated values of the separation factor, based on Abraham’s equation, are significantly different (*α*_ACN_ = 0.78; *α*_THF_ = 0.71) from those based on experimental data. Molecules of these solutes differ in lipophilicity, and this parameter is not included in the equation for calculating their retention. When *k* values are obtained using the Abraham equation with a supplementary term containing log*P*, *α* values are 1.76 (the system with ACN) and 1.88 (the system with THF), so the results match the experimental data better.

However, an increase in the 1-nitropropane retention relative to that of butan-1-ol in the THF system (*α* = 4.47) in comparison to the ACN system (*α* = 3.63) cannot be explained by differences in their hydrophobicity (log*P*_1-nitropropane_ = 0.87, log*P*_butan-1-ol_ = 0.88 [27]) and/or values of their molecular volume parameter (*V*_1-nitropropane_ = 0.71, *V*_butan-1-ol_ = 0.73) in spite of the fact that butan-1-ol shows proton-donor properties and 1-nitropropane does not. The retention increase of nitro derivatives cannot be explained with respect to dipolar or hydrogen bond interactions of the solutes with the modifiers in the stationary phase region (parameter *α* is even higher in the THF system than in the ACN system). The relative retention increase of the nitro derivative in the THF system in comparison to the ACN system can be explained in a similar way to that demonstrated for aromatic hydrocarbon derivatives with nitro group/s [6]. The nitro group forms a quadrupole. Two bonds between nitrogen and oxygen atoms in the nitro group are highly polarized with an electron density deficiency on the nitrogen atom [24]. On the other hand, the THF molecule has two C–O bonds (ether group), which are polarized with the higher electron density on the oxygen atom, and in this way they form a quadrupole, too. Both quadrupoles (nitro and ether groups) match each other, which leads to stronger electrostatic interactions between nitropropan and THF than between 1-nitropropane and ACN.

### Methanol (MeOH) versus Tetrahydrofuran

**Fig. 2:**
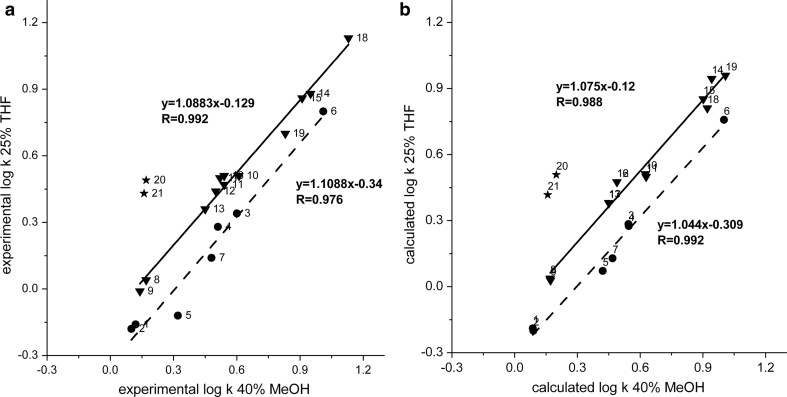
**a** Correlation of experimental log*k* values of aliphatic hydrocarbons with polar functional groups for the systems with 40 % MeOH and 25 % THF; *triangles* compounds with electron-donor group, *circles* alcohols, numbers of solutes, Table 1. **b** Correlation of calculated log*k* values of aliphatic hydrocarbons with polar functional groups for the systems with 40 % MeOH and 25 % THF; *triangles* compounds with electron-donor group, *circles* alcohols, numbers of solutes, Table 1

**Fig. 3:**
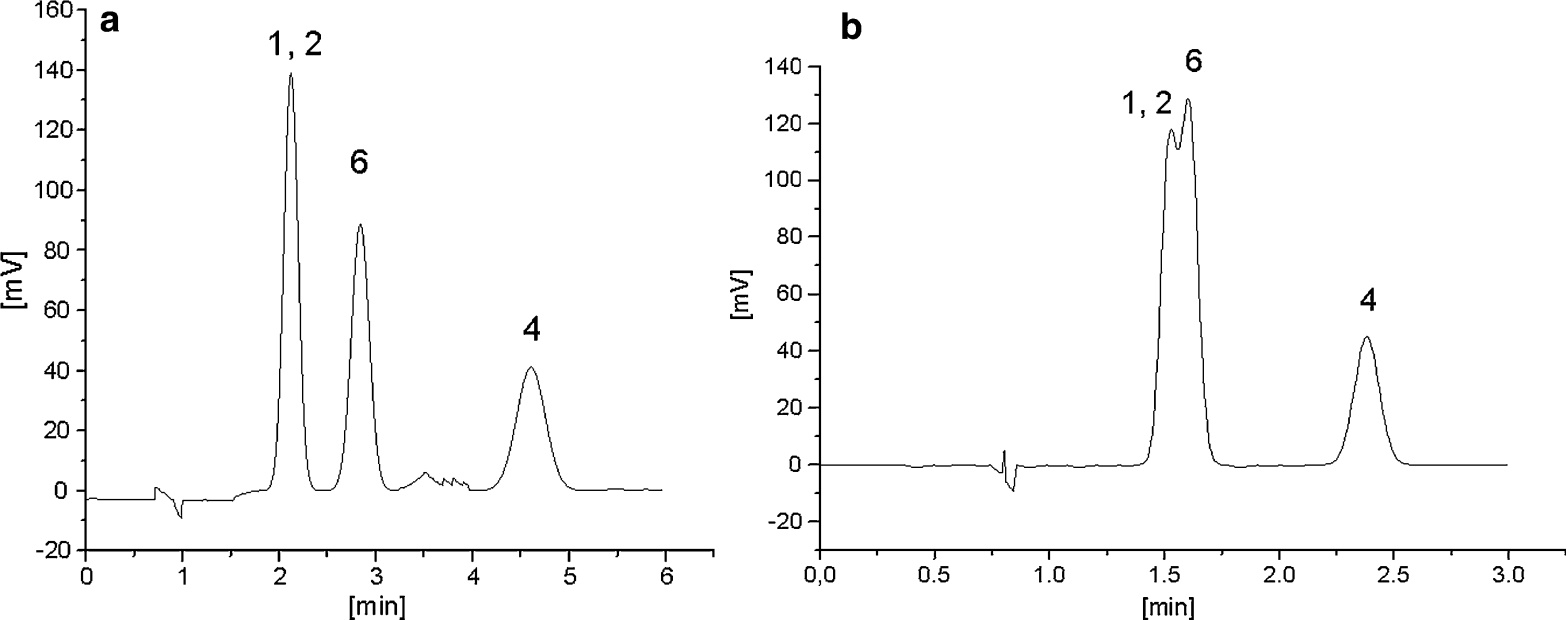
**a** Chromatogram of aliphatic hydrocarbons with polar groups, 40 % MeOH. Solute numbers in Table 1. **b** Chromatogram of aliphatic hydrocarbons with polar groups, 25 % THF. Solute numbers in Table 1

MeOH and THF differ significantly in their properties. MeOH has proton-donor ability, while THF could not act as a proton donor at all. MeOH has stronger proton-acceptor properties than THF, but its sorption on the stationary phase is several times smaller [17, 25]. Therefore, one might expect that alcohols, solutes with proton-donor and proton-acceptor properties, will show a retention increase in the THF system in comparison to the MeOH system, as was described above for the THF vs. ACN systems. However, inspection of the data in Fig. 2a leads to the observation that the correlation line for alcohols is located under the correlation line of solutes with electron-donor groups. This means that alcohols exhibit increased retention in comparison to the other substances in the MeOH system in relation to the THF system. It should be noticed that alcohols, donors and acceptors of protons, can strongly interact with the methanol contained in the stationary phase. Also formations of solvated species in the stationary phase region with self-associated methanol molecules and/or methanol/water complexes could lead to enhancement of their retention [26]. A similar effect was observed in our earlier studies of aromatic alcohols [6, 10]. The next example of relative retention changes is demonstrated for substances with a nitro group that exhibit a retention increase in comparison to the substances with a hydroxyl group in the THF system relative to that of MeOH: see for example 1-nitropropane and butan-1-ol (*α*_MeOH_ = 1.12, *α*_THF_ = 4.46). The explanation for this phenomenon is similar to the above-discussed ACN and THF systems. However, the effect of a retention increase in the case of nitro derivatives in the tetrahydrofuran system in comparison to the system with methanol is more pronounced than for the THF vs. ACN systems because methanol does not have such a large dipole interaction ability as ACN does. The separation factor values of the structural isomers (Table 3) show similar relationships as for the previously compared ACN—THF systems. The effect of a retention increase of the *n*-isomer toward branched iso-isomers is more explicit for the following pairs of substances: 1-propoxypropane and 2-propan-2-yloxypropane or 1-nitropropane and 2-nitropropane in the THF system in comparison to that in the MeOH system. A higher separation factor value is also observed for the isomer pair of pentan-1-ol and 2-methylbutan-2-ol in THF system, 2.88 (25 % THF), than in the MeOH system, 1.90 (40 % MeOH). However, the calculated values of the separation factor of the solute pair are equal to 1.33 and 1.63 for the MeOH and THF systems, respectively. This means that experimental selectivity of these two compounds in the MeOH and THF systems differs much more than that based on calculations using Eq. 1. The explanation for this difference seems to involve the solute molecules' lack of a shape parameter in Abraham’s equation, which could reflect various entropic solute penetrations of the stationary phase region in respect to its ordering by the extracted modifier. The relative retention changes of 2-methylbutan-2-ol additionally reflect different stationary phase ordering in the THF system relative to that of the MeOH system. As a result, 2-methylbutan-2-ol alcohol (log*P* = 1.08) shows comparable retention to butan-1-ol (log*P* = 0.88) and 2-methylpropan-1-ol (log*P* = 0.76) in the system with THF, despite the weaker hydrophobic properties of the last two substances (Fig. 3a, b).

**Table 3:** The values of separation factor *α* for the aliphatic hydrocarbon pairs with the same polar group (carbonyl, ether, and ester) in systems with different modifiers

	40 % MeOH	30 % ACN	25 % THF
Pentan-2-one/3-methylbutan-2-one	1.06	1.08	1.12
Hexan-2-one/4-methylpentan-2-one	1.17	1.10	1.09
Propyl acetate/1-methylethyl acetate	1.13	1.13	1.20
Butyl acetate/2-methylpropyl acetate	1.10	1.04	1.05
1-Propoxypropane/2-propan-2-yloxypropane	2.00	2.30	2.72
1-Nitropropane/2-nitropropane	1.03	1.06	1.13

A significant increase of separation factor for the ester—ketone pairs in the tetrahydrofuran system relative to the system with methanol could be noticed in the data presented in Table 4. As mentioned above, esters are more hydrophobic than ketones. The hydrophobic property of the stationary phase with THF is stronger than that with MeOH. Therefore, an increase of the relative retention of esters to ketones is observed in the THF system compared to the MeOH system. By analogy with the discussed effect, the higher separation factor values for the following pairs of solutes can be explained: pentan-1-ol and butan-1-ol, and 2-methylopropan-1-ol and 3-methylbutan-1-ol or hexan-1-ol and butan-1-ol in the THF system compared to those in the MeOH system (Table 5). The values of the separation factor of 1-propoxypropane/2-propan-2-yloxypropane are 2.0 and 2.9 in the system with methanol and tetrahydrofuran, respectively. Based on the calculated retention data (Eq. 1), the respective separation factor values are equal to: *α*_MeOH_ = 0.81; *α*_THF_ = 0.71. However, the separation factor values calculated with Abraham’s equation, into which the log*P* parameter was incorporated, yielded *α*_MeOH_ = 1.62 and *α*_THF_ = 1.86. Hence, the stronger hydrophobic character of the stationary phase region in the THF system in comparison to that of the MeOH system seems to be confirmed.

**Table 4:** The values of separation factor *α* for aliphatic hydrocarbon pairs with polar groups (ester and carbonyl) in systems with different modifiers

	40 % MeOH	30 % ACN	25 % THF
Propyl acetate/pentan-2-one	2.13	1.88	2.49
1-Methylethyl acetate/3-methylbutan-2-one	2.03	1.79	2.31
Butyl acetate/hexan-2-one	2.22	1.88	2.34
2-Methylpropyl acetate/4-methylpentan-2-one	2.37	1.99	2.43
Methyl butanoate/pentan-2-one	2.33	2.07	2.94
Methyl isobutyrate/3-methylbutan-2-one	2.38	2.20	3.19

**Table 5:** Values of separation factor *α* for aliphatic alcohols pairs in systems with different modifiers

	40 % MeOH	30 % ACN	25 % THF
Pentan-1-ol/butan-1-ol	3.02	2.22	3.16
3-Methyl-1-butanol/2-methylpropan-1-ol	2.54	2.27	2.85
Hexan-1-ol/pentan-1-ol	2.57	2.64	2.90

### Methanol versus acetonitrile

**Fig. 4:**
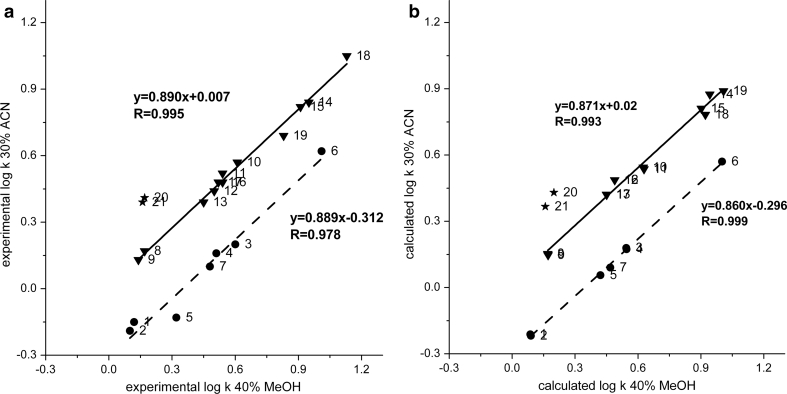
**a** Correlation of experimental log*k* values of aliphatic hydrocarbons with polar functional groups for systems with 40 % MeOH and 30 % ACN; *triangles* compounds with electron-donor groups, *circles* alcohols; solute numbers as in Table 1. **b** Correlation of calculated log*k* values of aliphatic hydrocarbons with polar functional groups for systems with 40 % MeOH and 30 % ACN; *triangles* compounds with electron-donor groups, *circles* alcohols, solute numbers as in Table 1

In Fig. 4a and b, the relationships log*k* ACN vs. log*k* MeOH are shown for experimental and calculated values of solute retention, respectively. The figures clearly demonstrate different separation selectivity of the solutes investigated between the MeOH and ACN systems. The correlation lines for alcohols are located below the correlation lines for the remaining compounds. This indicates that retention of alcohols increases relative to that of solutes without proton-donor properties in the system with MeOH in comparison to the system with ACN. Formation of strong complexes of alcohols with MeOH molecules and/or MeOH/water solvents in the stationary phase region may lead to an increase in their retention. In addition, methanol demonstrates proton-donor and proton-acceptor properties, which are responsible for its stronger propensity for H-bond formation with alcohols in comparison to ACN. It is worth noting that the correlation line for alcohols is much more below the correlation line for the remaining solutes compared to the analogous lines in Fig. 2a in which the THF vs. MeOH systems are demonstrated. This effect confirms the greater ability of MeOH to interact as a proton acceptor against aliphatic alcohols in comparison to THF, and especially ACN. This observation is also reflected in the value of the parameter *a* of the MeOH system, which is higher than that of the THF system and much higher than that of the ACN system (Table 2).

Significantly greater sorption of ACN in comparison to MeOH is responsible for the higher hydrophobicity of the stationary phase region of the chromatographic system with the former modifier. Hence, the separation factor for solutes of different polarities, e.g., 1-propoxypropane (log*P* = 1.13)/2-propan-2-yloxypropane (log*P* = 0.70), demonstrates higher values for the ACN system relative to that for the MeOH system (Table 3). However, the difference is not as significant as in the case of the MeOH vs. THF systems.

A significant increase in the retention of solutes with nitro groups relative to butan-1-ol (and other alcohols too) is noticeable in the system with ACN compared to the system with MeOH (*α*_ACN_ 1-nitropropane/butan-1-ol = 3.63, *α*_MeOH_ 1-nitropropane/butan-1-ol = 1.12). Nitro derivatives have rather large values of dipole moment, leading to higher retention in the system with ACN in comparison to the system with methanol.

The *α* factor values for the structural isomers in both systems (Table 3) are comparable. MeOH and ACN organize the stationary phase region to a lesser extent than THF does. Hence, the difference in the separation selectivity of solutes of different molecular shapes between these two systems is reflected to a lesser extent relative to the ACN vs. THF or especially MeOH vs. THF systems. Thes *α* factor value (Table 4) for ester and ketone pairs are higher in the system with MeOH in comparison to the system with ACN. This means that the retention of esters increases in comparison to ketones in the system with ACN relative to the system with MeOH. Ketones have greater dipole moment values compared to esters (the *S* parameter values for ketones are in the range: 0.65–0.68; for esters: 0.57–0.60). This could provide an explanation for the retention increase of ketones relative to esters in the ACN system in comparison to the MeOH system.

## Conclusions

The results presented in this article confirm the validity of our previously presented approach, which shows that the interpretation of separation selectivity changes (when one organic modifier of the mobile phase is replaced by another) could be performed taking into account (a) molecular interactions of the separated substances with the stationary phase components, especially the modifier, and (b) the ordering of the stationary phase, which depends on the type of mobile phase modifier. Hence, the molecular interactions of the solutes in the mobile phase could be neglected. Such an approach makes the interpretation of the results much easier—it takes into consideration molecular interaction in one phase, which simplifies optimization of RP HPLC separation conditions and makes them intuitive and understandable. The effect of the selectivity changes associated with the different degree of stationary phase ordering by the modifiers is especially important in the case of small molecules. The LSER can be applied in order to predict retention data, and such an approach could be helpful for explaining the selectivity changes of aliphatic compounds.

Our results suggest that the molecular interactions of solutes with the modifier extracted in the stationary phase play an important role in separation selectivity changes in RP-HPLC systems. Thus, a better understanding and quantitation of these interactions can lead to improvement of the prediction of separation selectivity changes with respect to the modifier choice.
